# Dr. Hamer's Report for the Year 1919

**Published:** 1920-08-28

**Authors:** 


### THE HEALTH OF LONDON.

### Dr. Hamer's Report for the Year 1919.

Dr. W. H. Hamer's report, as Medical Officer of Health and School Medical Officer for the County of London, is as important as the despatch of a Field-Marshal. It summarises the successes and failures of last year's campaign against the forces of disease. Disease is more lethal than bullets: during the influenza epidemic of 1918 more men were killed by that disease than by all the artillery thundering on the battlefields of Europe, while, as we know, the annual death-rate from tuberculosis in London alone is over 8,000. Disease, moreover, recognises no peace treaties and respects no national boundaries—a flea from Pekin may carry plague to Paris, a mosquito from Timbuctoo malaria to Tooting, a louse from Lemberg typhus to Lambeth—and Dr. Hamer's report is of international as well as national value.

### The Study of Multitudes.

A Medical Officer of Health is primarily concerned not with the health of individuals, but with the health of multitudes. The epidemic diseases naturally offer him most opportunity for broad surveys, but he is also specially concerned with the various environmental conditions which, even apart from infection, affect the general health of communities, and he has to consider such cognate questions as education, housing, and drainage.

Dr. Hamer handles his facts and his conclusions in a masterly way, and his survey of the field of battle shows that public health is scientifically testing old foundations and breaking new ground. His report is a record of devoted labour, of punctilious reasoning, and of radical reconstruction. To deal here adequately with the whole of the report is obviously impossible, and we must be content to mention some of its most salient features. Under "Vital Statistics" several interesting and rather surprising facts are pointed out. We learn, for instance, that, despite the lamentable lack of house accommodation in London, the population is probably less than before the war. Dr. Hamer attributes the house shortage to the high marriage-rate, necessitating the creation of separate establishments. The marriage-rate in the five years 1915-19 was 234,694, as compared with 206,421 in 1910-14. It is also probable, though Dr. Hamer does not mention it, that the rise in wages has led to some amount of exodus from the slums, and that less congestion there has meant more congestion elsewhere.

### The Birth-Rate.

Along with the increase in marriage-rate there has been a slight increase in birth-rate; but the birth-rate—18.2 per thousand—was in 1919 far below the rate of the last decades of the nineteenth century. The death-rate during the year 1919 is the lowest ever recorded, this happy fact being due chiefly to a remarkably low death-rate from measles and whooping-cough. From the former of these the death-rate was "far below any rate hitherto recorded in London, even when the diminution in the birth-rate has been fully taken into account." On the other hand, scarlet fever, which had been conspicuous by its comparative absence during the previous three years, became much more prevalent and diphtheria showed a considerable increase. The incidence of typhoid remained at the low level of the preceding few years.

Dr. Hamer devotes ten or eleven pages to a very interesting discussion of the source of infection in typhoid, and deals particularly with the question of infection by sewage-polluted fish, quoting in this connection some interesting passages from a Departmental Report on Inshore Fisheries. The conclusion Dr. Hamer reaches is that polluted water supplies, small ungutted flatfish, and shell-fish are the main causes of typhoid fever, and that the parts played by milk and by typhoid-carriers have been rather exaggerated. He makes the suggestion that perhaps, epidemiologically considered, milk-typhoid may not be quite identical with water, and fish, and shell-fish typhoid, and that "we are apt to fail to recognise that symptom-complexes may result from the action of more agencies than one, each of them competent to set in motion the required sequence of events and thus to serve as a link in the chain of causation in question."

### Tuberculosis Records.

The report on tuberculosis is satisfactory, since the deaths from pulmonary tuberculosis in the civil population during 1919 were only 5,332, as compared with 6,491 in 1916 and 6,908 in 1917. Dr. Hamer doubts the interpretation put by Dr. Gordon on his statistics of the phthisis mortality in the exposed and sheltered districts of Okehampton. Dr. Gordon's inference that exposure to the south-west or west wind is conducive to phthisis was plainly unsound, for an exposed situation may mean less agricultural prosperity or less window ventilation, or various other things, each sufficient without direct intervention of the wind to promote phthisis.

In the course of some interesting pages on in-influenza, Dr. Hamer suggests that influenza, cerebro-spinal fever, poliomyelitis, and polioencephalitis (including encephalitis lethargica) are one disease assuming different types according to "the degree and extent to which the particular population concerned is exposed to infection on the one hand, and susceptible or immunised by previous attack on the other." That is at least an interesting theory and shows the almost revolutionary courage of modern epidemiological science. The latest work on the ultra-visible viruses and their possible mutations is referred to in this connection, and mention is made of the extraordinary experiments made by Dr. Milton J. Rosenau and others to determine the infectivity of influenza. Many volunteers offered themselves for experiment, and every effort was made to infect them with influenza. Their nostrils, throat, and eyes were sprayed with bacilli—some

**The Health of London.**—(*continued*).

of the bacilli being obtained from the lungs of dead influenza patients. Their nostrils, throat, and eyes were smeared with secretions from the mucous membranes of influenza cases; blood from influenza patients was injected; and still other methods were used to infect them.

The results were mainly negative. Some of the volunteers contracted tonsilitis, and two or three had febrile attacks resembling influenza; and so far as the experiments proved anything they merely proved that influenza is not very infectious, and does not so readily spread from case to case as has been hitherto generally supposed.

Health in Schools.

Dr. Hamer's report, as School Medical Officer, is no less interesting and important than his report as Medical Officer of Health. Disease in adult life is often the result of hygienic delinquencies and hygienic transgressions in childhood; and the future adult vigour of a nation must depend on the health of its children. The deplorable crop of unfit revealed by recent recruiting statistics was largely the result of unfavourable influences operating in childhood. It is good, therefore, to read Dr. Hamer's optimistic report of the magnificent work which is being done among school children—work that, in the opinion of all those qualified to judge, is already beginning to bear good fruit. It is good, too, to know that all through the war the hygienic condition of the children continued to improve year by year. In 1913, 10.8 per cent. of entrant boys were undernourished; in 1918, 4.7 per cent. In 1913, 15.8 per cent. of the eight-year-old group of boys were underfed; in 1918, 3.3 per cent. In 1913, 5.5 per cent. of the boy "leavers" were undernourished; in 1918, 3.6 per cent.; while in clothing and cleanliness there was corresponding improvement. In 1919 there is some deterioration in the nutritional condition of the children in all groups; but it is still better than in pre-war days, and the deterioration is probably due to the increased price of food.

During the year 1919, 195,162 children were inspected and 155,020 re-inspected; and 28,000 eye cases, 12,837 ear, nose, and throat cases, 1,322 ringworm cases, 12,000 minor ailment cases, and 79,216 dental cases were treated. These figures alone suffice to indicate the colossal amount of work that is being done.

But even these cover only a small part of the work reported by the School Medical Officer. The infectious diseases, of course, are in his purview, and in schools infectious diseases are particularly prevalent. There were 5,574 cases of scarlet fever, and "228 special investigations were conducted at schools by the Council's medical staff." There were 3,365 cases of diphtheria, and 116 special investigations were made, and 2,924 throat, nose, and ear specimens were cultivated and examined in the laboratory. The inquiries made in previous years into the relationship between fleas and scarlet fever were continued in 1919, and tended to confirm the idea of some causal connection.

In the section reporting on the infectious diseases Dr. Hamer has a very interesting discussion on the periodicity, and on the variation in type of the infectious diseases. The question is important, for any light on the reasons of the periodicity and varying virulence of the infectious diseases must also throw light on their whole ætiology. Dr. Hamer is of the opinion "that variation of type in disease depends on environment (including in this term the several degrees of immunisation of affected populations), as well as upon changes in disease germs; and, further, as regards the latter, it is clear that the searchlight of inquiry must not be merely focused upon the bacteria which have hitherto formed the main object of interest, but must be even more particularly directed to the study of cyclical changes in the life-history of far more elusive organisms."

Other sections of Dr. Hamer's report as School Officer deal with such important matters as school meals, nursery-schools, open-air education, physical training, personal hygiene, school baths. From first to last the double report is not only a record of magnificent work, but also a mine of useful sociological and scientific information, and the various diagrams included in the report greatly add to its value.

